# Characteristics and significance of peripheral blood T-cell receptor repertoire features in patients with indeterminate lung nodules

**DOI:** 10.1038/s41392-022-01169-7

**Published:** 2022-10-10

**Authors:** Huaichao Luo, Ruiling Zu, Ziru Huang, Yingqiang Li, Yulin Liao, Wenxin Luo, Peng Zhou, Dongsheng Wang, Shifu Chen, Weimin Li, Jian Huang

**Affiliations:** 1 School of Life Science and Technology, University of Electronic Science and Technology of China, Chengdu, Sichuan China; 2 Department of Clinical Laboratory & Division of Radiology, Sichuan Cancer Hospital & Institute, Sichuan Cancer Center, School of Medicine, University of Electronic Science and Technology of China, Chengdu, Sichuan China; 3 HaploX Biotechnology Co., Ltd, Shenzhen, China; 4 Department of Respiratory and Critical Care Medicine, West China Hospital, West China Medical School, Sichuan University, Chengdu, 610041 China

**Keywords:** Lung cancer, Predictive markers

**Dear Editor,**

Lung cancer is one of the most common cancers and a leading cause of cancer-related deaths. T cells are known to play a significant role in the destruction of cancer cells. T cells have therefore become the focus of lung cancer immunotherapy. T-cell receptors (TCRs) can recognize antigenic peptides presented by HLA proteins. TCRs are distinct individually and vary with pathophysiological condition, so T cells can respond to a wide range of antigens. TCR repertoire diversity reflects the potential for cellular immunity, and several studies have demonstrated that complementarity determining region 3 (CDR3*β*) diversity is important in cancer therapy and prognosis.^1^ Liu et al. have reported that the CDR3*β* diversity of patients with advanced lung cancer differs significantly from that of healthy individuals.^2^

Here, we have developed a novel model called “TCRnodseek” based on the repertoire properties of TCRs in peripheral blood, which can accurately classify small pulmonary nodules into malignant or benign types. The prospective study was initiated at Sichuan Cancer Hospital (Supplementary Materials). The flow chart for this study is shown in Fig. 1a (Supplementary Fig. S1). In Supplementary Table 1, we describe the main baseline characteristics of the 109 patients. Among them, 99 patients with indeterminate lung nodules are the main research subjects. The number of male and female subjects is comparable (male 52; 52.5%), the mean age is 55.5 years, and the mean size of indeterminate lung nodules is 13.7 mm. There are two independent groups of patients enrolled in this study (a discovery group and a validation group). We extracted DNA from their peripheral blood samples and analyzed their TCR repertoires. Supplementary Table 2 provides detailed information on the quality control of sequencing.

**Fig. 1:**
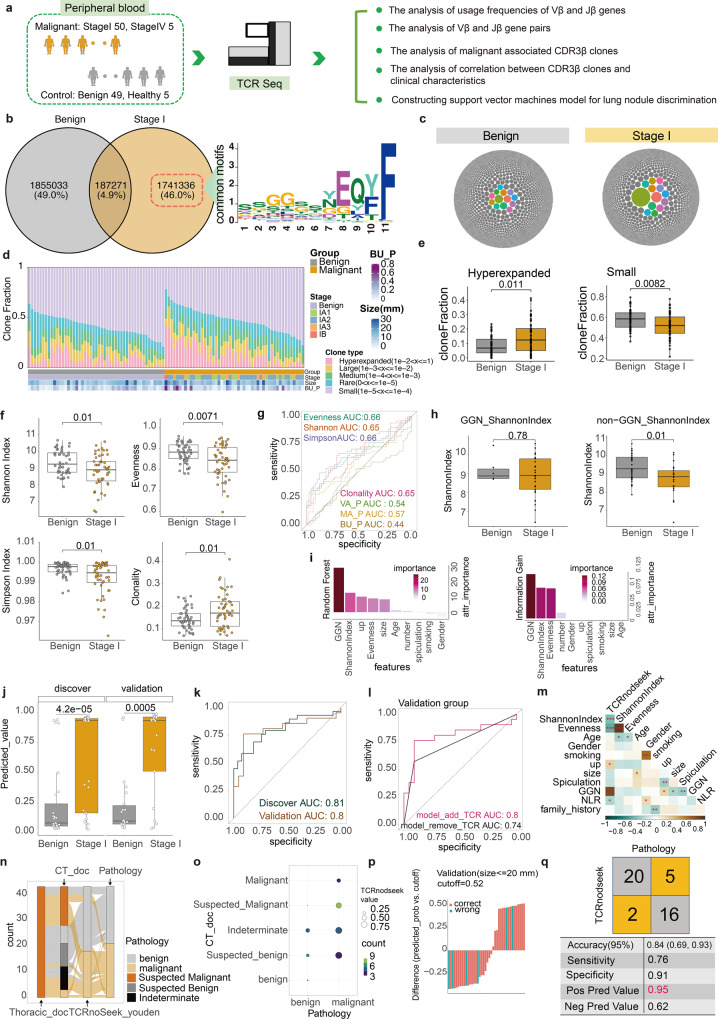
The study design and main results. **a** In this prospective cohort study, patients suspected of having indeterminate pulmonary nodules were enrolled and their CT information was recorded. Blood samples were collected and stored prior to surgery. TCR sequencing was performed and analyzed. **b** Venn diagrams were used to identify clones just existing in the malignant group. common motif of enriched CDR3β aaSeqs is displayed. **c** The top 10,000 TCR clones are shown by Packed circles for the benign and stage I groups (one circle represents one clone; circle size represents clonal fraction). **d** The relationship between the clone types and the clinical characteristics of the benign and malignant groups is demonstrated. **e** A boxplot to illustrate the difference between benign and malignant clones for small-type and hyperexpanded-type clone types. **f** Comparison of TCR repertoire diversity between benign and malignant groups using boxplots. **g** Comparison of the diagnostic value of TCR repertoire diversity with previous models using ROC curves. **h** Boxplot showing the difference in Shannon index between benign and malignant groups in the GGN group and the non-GGN group separately. **i** Random forest and information gain methods were used to select significant features for the TCRnodseek model. **j** The predicted value calculated by TCRnodseek in the discovery and validation groups are presented as boxplots and ROC curves (**k**). **l** the ROCs of the models with and without TCR features. **m** the relationship between TCRnodseek predicated value and various clinical characteristics. **n** A Sankey diagram illustrating the misclassification of TCRnodseek and experts in comparison with pathology results (Thoracic_doc, the results determined by Thoracic doctors; CT_doc, the results determined by Radiology doctors; TCRnodseek_youden, the results determined by TCRnodseek with youden cutoff). **o** A bubble plot illustrating the relationship between CT doctors’ diagnoses, pathology results, and TCRnodseek predicted value (the size of the bubble represents the mean predicted value of TCRnodseek; the color of the bubble represents the number of subjects in the condition). **p** A waterfall plot showing the TCRnodseek function for nodule sizes <= 20 mm (the X-axis represents the sample id sorted by the predicted value of TCRnodseek; the Y-axis represents the predicted value of TCRnodseek minus the cutoff value). **q** The diagnostic performance of TCRnodseek in the validation group (the original subjects of (**b**) to (**i**) figures are all subjects; the original subjects of (**l**) to (**q**) figures are independent validation subjects; GGN ground glass nodule)

Utilizing Venn diagrams, we identified malignant associated clones (46% of all clones) that only existed in the stage I group (Fig. 1b). We defined the top 30 CDR3 amino acid sequences and the top 3000 sequences that are present in at least two subjects are enriched CDR3*β* amino acid sequences (aaSeqs, Supplementary Fig. S4). In enriched CDR3*β* aaSeqs, one common motif was observed, ‘SSGGSSYEQYF’, which is similar to some previously reported high-quality specificity CDR3*β* aaSeq motif in non-small cell lung cancer^3^ (Supplementary Data S1, Data S2). In addition, some CDR3*β* aaSeqs were annotated by VDJdb, which matched multiple HLA types and antigens. Then, we applied pMTnet to rank various pairs and obtained three candidate CDR3*β* aaSeqs (Supplementary Data S1).^4^

The correlation between clonal fraction and clinical characteristics was studied. As shown in Fig. 1c, packed circles indicate that some stage I subjects harbor high fraction clones. We classified TCR clones into five types: hyperexpanded, large, medium, small, and rare clone types (Fig. 1d). The benign group harbored significantly more small-type clones, whereas the malignant group harbored significantly more hyperexpanded-type clones (*p* = 0.0082 and *p* = 0.011, respectively, Fig. 1e). The relationship between TCR features and clinical information was assessed using the spearman correlation. The correlation plot reveals that nodule size (largest diameter) is positively correlated with CloneReads (*p* = 0.01, *r* = 0.24, Supplementary Fig. S3).

The Shannon index, evenness index, Simpson index, and clonality index were significantly different between the benign and stage I group (*p* = 0.01, *p* = 0.0071, *p* = 0.01, *p* = 0.01, respectively; Fig. 1f, Supplementary Data S3). ROC analysis was used to determine the diagnostic value of the above features, and the AUC of each feature was higher than that of previous nodule diagnosis methods (Fig. 1g). In addition, we have added several important clinical features for the diagnosis of lung nodules to the list of candidate features. Random forest and information gain methods were applied to select the top three vital features (Ground glass nodule, Shannon index, and evenness index) to distinguish benign from malignant lung nodules (Fig. 1i). GGN (Ground glass nodule) is the most important feature. The Shannon index can be used as a useful complementary indicator for non-GGN indeterminate lung nodules (*p* = 0.01, Fig. 1h), which is consistent with the previous study.^3^

Next, a robust support vector machine model was constructed and its parameters were optimized with genetic algorithm (Supplementary Fig. S5). The model was thereafter called TCRnodseek, which was short for seeking to distinguish malignant nodules from benign ones via a TCR-based model. TCRnodseek performed well in both discovery and validation groups (AUC values of 0.81 and 0.80, respectively; Fig. 1j, k). In the validation group, the AUC value of a model without TCR features decreased to 0.74 (Fig. 1l). The correlation between TCRnodseek predicted value and clinical information was determined using spearman correlation (Fig. 1m).

Furthermore, our model was able to correctly identify not only suspected malignant lung nodules diagnosed by a thoracic surgeon, but also most indeterminate lung nodules determined by an advanced radiologist with more than 10 years of experience (Fig. 1n, o, Supplementary Fig. S4). In addition, TCRnodseek was highly accurate for indeterminate lung nodules less than 20 mm or 10 mm in size (Fig. 1p and Supplementary Fig. S6). TCRnodseek performs exceptionally well in the validation group, with a positive predictive value of 0.95 (Fig. 1q).

Our study found the highest TCR diversity in benign subjects, which is in agreement with results reported in a renal cell carcinoma study.^5^ In addition, we have applied bladder cancer and bladder benign data to validate,^6^ and we found that the TCR Shannon index of cancer is lower than that of benign subjects (Supplementary Fig. S7). In stage I subjects, we observed a decrease in TCR diversity, which may be related to intratumoral T cells with clonal expansion that circulated to the peripheral bloodstream.^7^ In contrast to stage II and stage III, stage I subjects possess a significantly higher diversity of TCRs^8^ (Supplementary Fig. S7). In this way, TCRnodseek may be used easily to detect malignant lung nodules with stage II and stage III as positive lung nodules.

Currently, regular low-dose CT (LDCT) screening is the most effective way to detect lung cancer early. LDCT screening, however, has a false positive rate of 96.4%.^9^ Experienced physicians can exclude some benign nodules using image features and clinical characteristics. Nevertheless, 30–50% of patients undergoing surgery are still diagnosed as benign nodules pathologically in the end. In fact, regular screenings and unnecessary surgeries have resulted in unnecessary medical care and psychological stress. Liquid biopsy may help. However, the AUCs of published liquid biopsy methods are <0.85 in validation groups.^10^ TCRnodseek provides a promising solution to this issue, with AUC of 0.8. It depends on three features: GGN, Shannon index, and Evenness. The first feature can be obtained from LDCT, and the two left features can be computed from TCR repertoire data of a patient. To obtain TCR repertoire data, only about 1 ml of peripheral blood is needed, which can be very easily got in clinical practice.

A web server, named “Tool Box of Lung Nodule Predictors” (TB-LNPs) (http://i.uestc.edu.cn/TB-LNPs), has been developed to allow public access to the TCRnodseek model. Also, we collected several other canonical models, which could be used in academic studies to evaluate indeterminate lung nodules.

In conclusion, we have developed the TCRnodseek model, which integrates TCR diversity and clinical information to distinguish benign from malignant lung nodules more accurately. In addition to providing evidence for diagnosis, information on CDR3*β* might benefit the development of CAR T-cell Therapy.

## Supplementary information

Supplementary_Materials

Data S3

Data S1

Data S2

Data S4

## Data Availability

Raw data of this project have been uploaded to https://bigd.big.ac.cn/gsa (HRA001754 and HRA002253).
