# Supplementary material for: Characteristics and significance of peripheral blood T-cell receptor repertoire features in patients with indeterminate lung nodules
Source: Signal Transduct Target Ther. 2022 Oct 10;7:348. doi: 10.1038/s41392-022-01169-7 (PMC9548502; doi:10.1038/s41392-022-01169-7)
Supplement: Supplementary file 1 — Supplementary_Materials [file 41392_2022_1169_MOESM1_ESM.docx]

Supplementary Materials for

**Characteristics and significance of peripheral blood T-cell receptor repertoire features in patients with indeterminate lung nodules**

Huaichao Luo, Ruiling Zu, Ziru Huang, Yingqiang Li, Yulin Liao, Wenxin Luo, Peng Zhou, Dongsheng Wang, Shifu Chen, Weimin Li, Jian Huang

Correspondence to: [hj@uestc.edu.cn](mailto:hj@uestc.edu.cn)

**This PDF file includes:**

Materials and Methods

Supplementary Text

Figures. S1 to S8

Tables S1 to S2

Captions for Data S1 to S4

**Other Supplementary Materials for this manuscript include the following:**

Data S1 to S4

Data S1. (separate file): Clone Fraction, annotation of all candidate TCR clones and HLA

genotyping data

Data S2. (separate file): The raw data of common motifs identified by MEME tool

Data S3. (separate file): TCR diversity information of main enrolled subjects

Data S4. (separate file): V/J gene segment usage data of main enrolled subjects

Materials and Methods

**Study design and participants**

This prospective cohort study was initiated in 2021 in Sichuan Cancer Hospital. The study was approved by the medical ethical committee of Sichuan Cancer Hospital (SCCHEC-02-2021-037). Subjects with a lung nodule diagnosed as suspected lung cancer from Thoracic Surgery in Sichuan Cancer Hospital were enrolled in the study. All of the included patients were required to meet the following criteria: (1) pathologically and immunohistochemically confirmed diagnosis by expert pathologists; (2) sufficient clinical data; (3) a lack of acute infection or chronic active inflammatory disease. Clinical information (age, gender, smoking history, anamnesis, family history, and personal history), computed tomography information (nodule size, number, characteristics, and position), pathology information (pathological type and stage), and laboratory information were captured and summarized. After obtaining pathological results, the subjects with benign and malignant cancers were enrolled for TCR sequencing. Peripheral blood was collected before surgery and stored at −80°C until use. Clinical information was collected from the hospital management system. To validate the application value, a CT expert with 10 years’ experience in diagnosing lung nodules was invited to evaluate all the enrolled subjects again. The radiology expert was blinded when re-evaluating the CT results to five levels (benign, suspected benign, indeterminate, suspected malignant, and malignant). To avoid the influence of a priori knowledge in the experimental process, the technical staff was blind to the study design and had no prior knowledge of lung cancer diagnosis. After the prospective sample collection, the sample sizes of the benign and malignant groups were balanced. To validate the biological significance of the peripheral blood TCR repertoire features, five normal (healthy) controls from the testing center and five patients with confirmed metastatic lung adenocarcinoma were included in this study. In Sichuan Cancer Hospital, the pulmonary nodules were detected by SIEMENS Definition Flash CT (SIEMENS Healthineers Co., Ltd, Erlangen, GER) and PHILIPS Brilliance iCT (Koninklijke Philips N.V.， Amsterdam， NED).

**High-throughput sequencing of TCR-β genes**

High-throughput sequencing of TCR-β genes was performed using previously described methods^1^ Briefly, we extracted total genome DNA from peripheral blood. The TCR-β DNA was amplified using multiplex PCR. Sequencing libraries were loaded onto the Illumina NavaSeq6000 System.

For quality control, we applied fastp as a FASTQ preprocessing tool and obtained clean fastq data ^2^. The MiXCR software package was used to map reads to the CDR3 sequence ^3^ and VDJtools was used to analyze the CDR3 sequencing ^4^. Shannon’s entropy was calculated based on the clonal abundance of all productive TCR sequences; the normalized Shannon’s entropy (Shannon index) was determined by dividing Shannon’s entropy by the natural logarithm of the number of unique productive TCR sequences. Several diversity indexes (Shannon index, Simpson index, evenness index, and clonality) were calculated to reflect the diversities of TCRs. The formulas of these indexes were as follows:

Shannon index = $-\sum_{i=1}^{n} pi\times lnpi$;

Simpson index = 1-$\sum_{i=1}^{n} pi^2$;

Evenness index = ($-\sum_{i=1}^{n} pi\times lnpi$)/$\ln n$;

Clonality = 1 + ($\sum_{i=1}^{n} pi\times lnpi)/\ln n$.

In these formulas, $n$ represents clone type counts,$i$represents one specific clone type, and $pi$ represents the reads for $i$, which were divided by all read counts. In this study, CloneReads represent all CDR3 clone read counts in one subject sample, whereas CloneCounts represent unique CDR3 clone counts in one subject sample. Three canonical lung cancer diagnosis models were developed using available data, including, the Brock University (BU) model^5^, the Mayo Clinic (MC) model^6^, Veterans Affairs (VA) ^7^ model are calculated, which are available in “Tool Box of Lung Nodule Predictors”.

**HLA genotyping**

HLA typing was carried out by polymerase chain reaction sequence specific primer (PCR-SSP) (MicroSSP^TM^ Generic HLA Class I and Class II ABDR DNA Typing Tray 384- SSPABDR, One Lambda Inc., Canoga Park, CA or Biotest ABDR SSP tray cat no. 826230, Biotest AG, Germany).

**Statistical analysis**

Continuous variables are displayed as means (±standard deviation), whereas discrete variables are displayed as count data (percentages). All statistical analyses were conducted in R (version 4.0.3). Mann-Whitney U tests were applied for comparisons of two groups. The ggplot2 R package was used for data visualization. Correlations between variables were analyzed using Spearman correlation analysis. Random forest and information gain were applied to selected top vital features using the caret R package. Genetic Algorithms are applied by the GA R package. 10-fold cross-validation is applied to Genetic Algorithms. root-mean-square deviation (RMSD) is applied to evaluate the model in 100 generations of Genetic Algorithms.

In addition, a support vector machine was constructed using selected features with the e1071 R package. p-value < 0.05 was considered statistically significant on a two-sided basis. We have performed multiple comparison adjustment by false discovery rate (FDR) adjust. Packed circles are drawn by packcircles R package. The top 10000 clone counts for each group are selected for packed circles. For the coexist analysis, we obtained unique CDR3β amino acid sequencing (aaSeqCDR3) from different groups, then we obtained malignant unique aaSeqCDR3.

Supplementary Text

**High-throughput sequencing of Vβ and Jβ fragments**

TCRs are composed of *Vβ* and *Jβ* fragments. The frequency of four *Vβ* genes and two *Jβ* genes was significantly higher in the benign group than in the stage I group, especially for TRBV10-2 (p value = 0.0064) and TRBJ2-2 (p value = 0.021), however, after FDR adjustment, all adjusted p values are greater than 0.05 (supplementary Fig1, supplementary Data S4). between contrast, the usage of other *Vβ* and *Jβ* genes was largely similar in the two groups. The distribution of Vβ and Jβ genes in different stages and nodule size groups is shown as a heatmap (supplementary Fig S2). Afterward, the *Vβ* and *Jβ* gene pairs were analyzed, revealing differences in the frequency of usage between the malignant and benign groups. In supplementary Figure S8, the top 20 V*β*-J*β* paired genes are depicted; most of the paired genes were downregulated in stage I patients. TRBV6-3-TRBJ2-6 pair is significantly more prevalent in the malignant group, however, after false discovery rate (FDR) adjustment, all adjusted p values are greater than 0.05.

We found that the clonal fraction of TRBV10-2 in the benign group was significantly higher than that in stage I malignant group (supplementary Fig S2). TRBV10-2 (T Cell Receptor Beta Variable 10-2) is a protein-coding gene, which is predicted to be involved in the cell surface receptor signaling pathway, a component of the T cell receptor complex, and active in the plasma membrane. Chen et al. reported that the TRBV10-2 segment was significantly more common in non-tumor tissues8. Additionally, TRBV10-2 is a major clonotype of one benign and asymptomatic disease (Chronic idiopathic neutropenia)^9^. For *Vβ*–*Jβ* pairs, TRBV6-3 combination with TRBJ2-6 frequencies significantly differed between the case and control. As far as we are aware, this is the first instance of a *Vβ*–*Jβ* pair in the peripheral blood of a lung cancer patient.

**The investigations of** CDR3*β* **amino acid sequences**

The unique amino acid sequences count of CDR3*β* clones in the benign group were similar to that of the stage I group (p = 0.36; supplementary figure S3). We defined the top 30 CDR3*β* amino acid sequences and the top 3000 sequences that are present in at least two subjects are enriched CDR3*β* amino acid sequences (supplementary Data S1**)**. The majority of CDR3*β* amino acid sequences are novel after being investigated in silico by three major TCR specificity databases (VDJdb, McPAS-TCR, and TCRMatch) ^10-12^. In silico, CASSIFGEQFF is predicted to be a Human Cytomegalovirus-specific CDR3*β* amino acid sequence by VDJdb tools. Most of other CDR3*β* clones are annotated by TCRMatch with cutoff of 0.9. Then, we used MEME to identify common motifs, and found one common motifs (Fig 1b) ^13^

If we do not consider clone fraction, all CDR3*β* aaSeqs that were annotated by VDJdb, McPAS-TCR were included in the supplementary data S1. CATSDPGQGTTGELFF is the highest fraction clone, which coexisted in two individuals. The HLA genotypes (HLA-A, HLA-B, HLA-DR) of these two subjects were measured, but all genotypes and HLA-A superfamilies were inconsistent **^14^**. Some CDR3*β* aaSeqs were annotated by VDJdb, which matched multiple HLA types and Antigens. We applied pMTnet to rank various pairs for three top CDR3*β* aaSeqs (supplementary Data S1**)^15^**.

**The potential limitation of this study**

Due to the small sample size, we are unable to draw definitive conclusions for both metastatic and healthy participants. Nevertheless, using the immunoSEQ Analyzer, we reviewed public data and found that TCR diversity is similar in metastatic and healthy individuals (supplementary figure S5). To further validate these special results, we have registered a clinical trial with the Chinese Clinical Trial Registry (ChiCTR2200055761).

In view of the limitations of this study as a prospective case-control study, additional well-designed prospective cohort studies are required to confirm our findings. However, for the TCRnodseek model, we have applied absolute independent two groups to construct and validate the model. Additionally, we utilized two independent methods to evaluate feature importance. By doing so, we have avoided overfitting to some extent. The TCR diversity index is indeed an important feature in the diagnosis of indeterminate lung nodules. Furthermore, HLA information is not available for most samples, which makes it difficult to detect tumor-specific antigens.


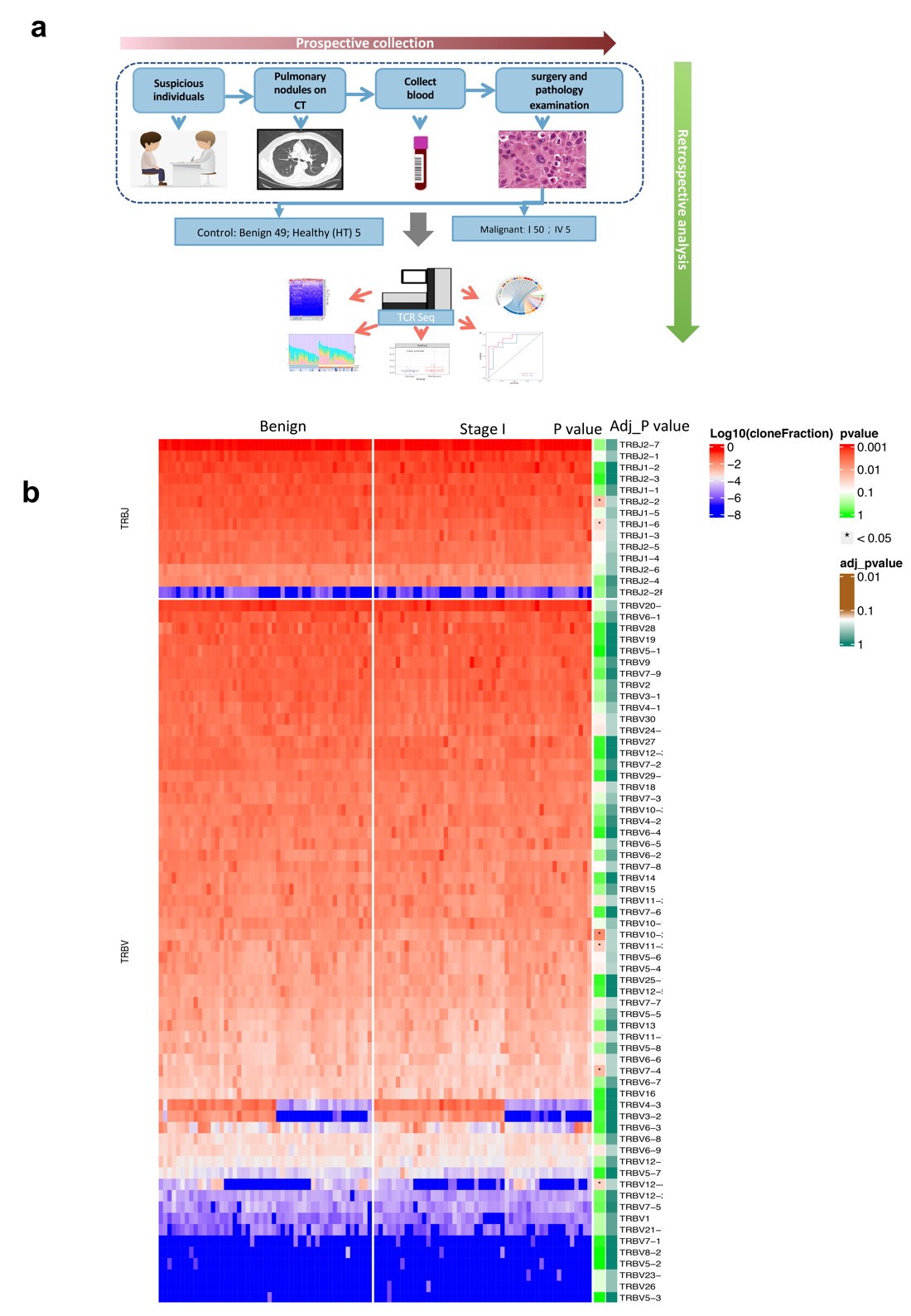


Figure. S1. Design of the study and the use of the *Jβ/Vβ* gene

The study design (a) and the usage frequencies of *Vβ* and *Jβ* genes are displayed in a heatmap. The heatmap is based on log10 transferred frequencies of *Vβ* and *Jβ* genes (b).


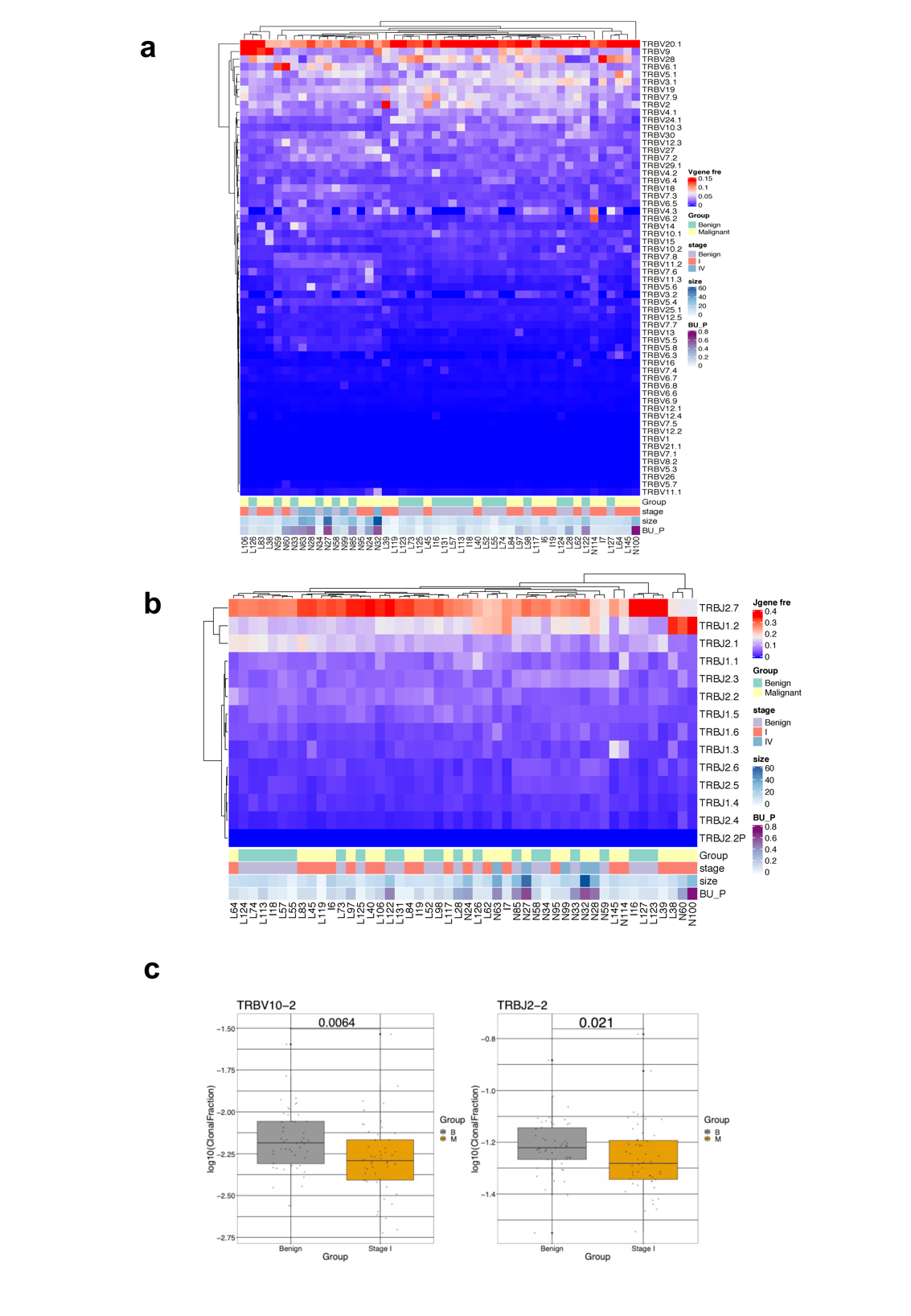


Figure. S2. the use of the *Jβ/Vβ* gene and clinical characteristics.

Usages of (a) the *Jβ* gene and (b) the *Vβ* gene in the distribution of different stage and nodule size groups are shown in heatmaps with raw frequencies of *Vβ* and *Jβ* genes. (c) The boxplots show two representative genes that are distributed between benign and stage I tumors.


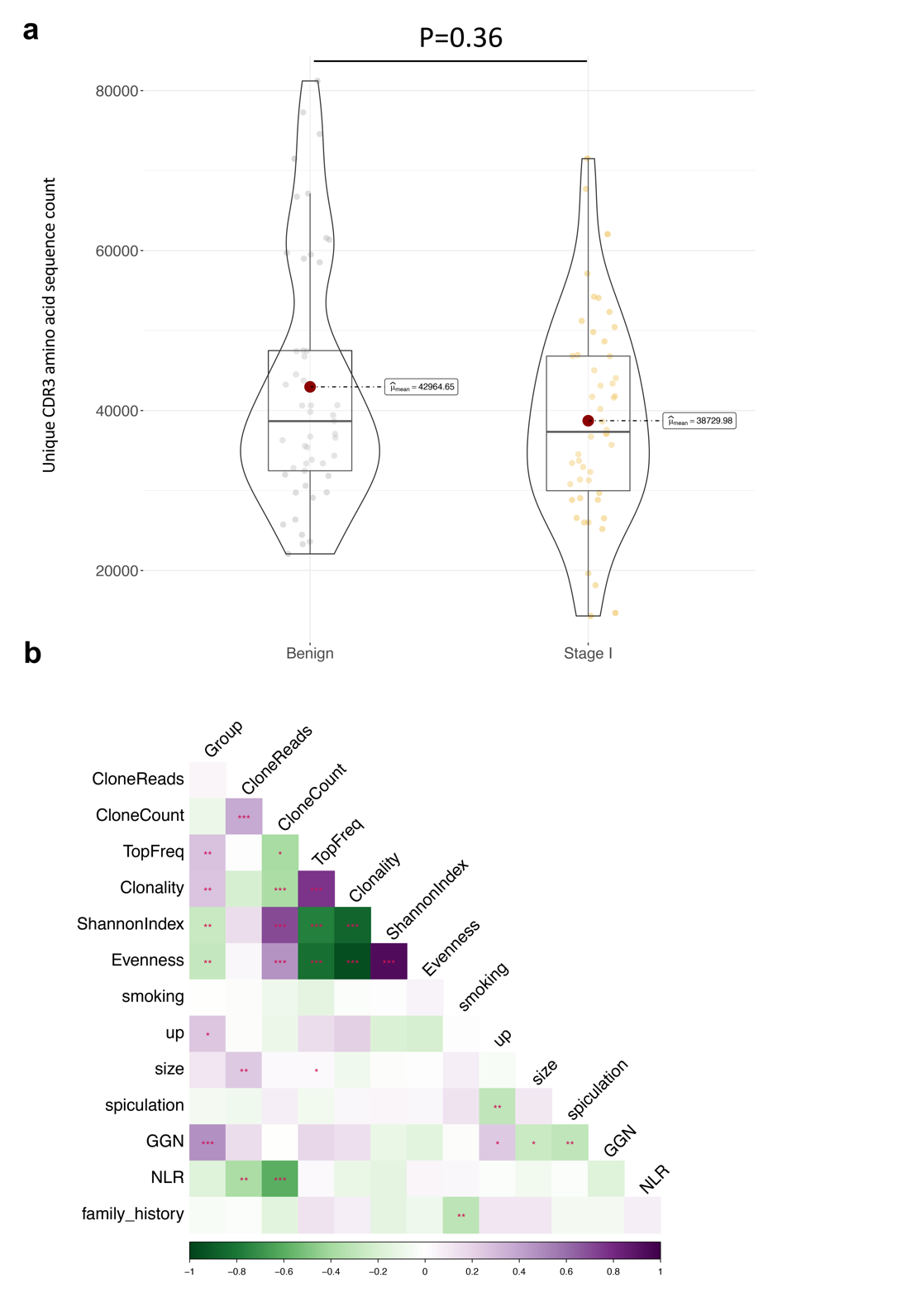


Figure. S3. Relationship between TCR clone features and clinical characteristics.

(a) The unique CDR3 amino acid sequence count of benign and malignant tumors was compared. (b) The relationship between TCR clone features and clinical characteristics.


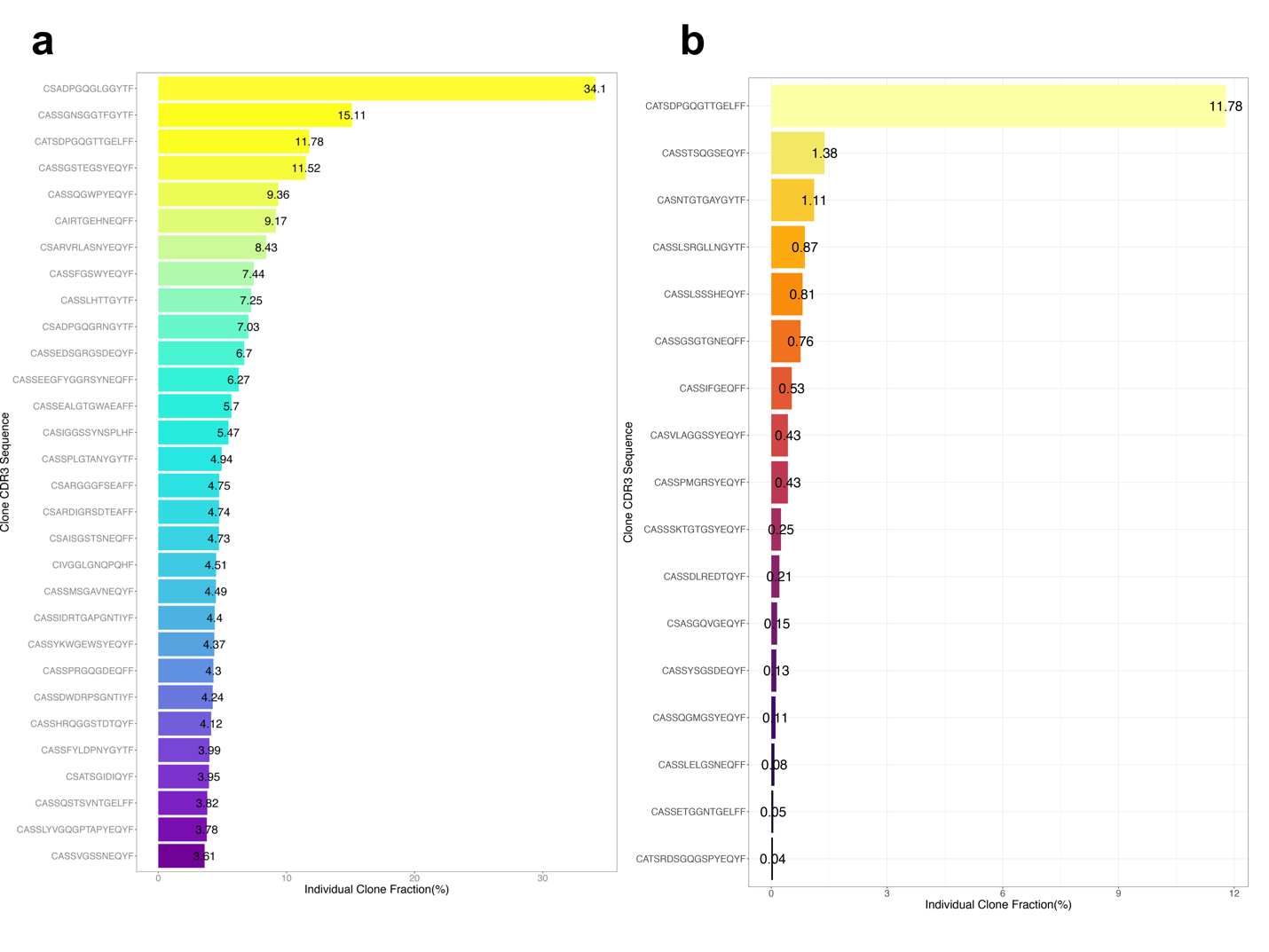


**Figure. S4. The bar plot of top high fraction TCR clones**

(a) Top30 high fraction TCR clones are shown in a bar plot. (b) Top1000 high fraction TCR clones and coexisted at least two subjects are displayed in a bar plot.


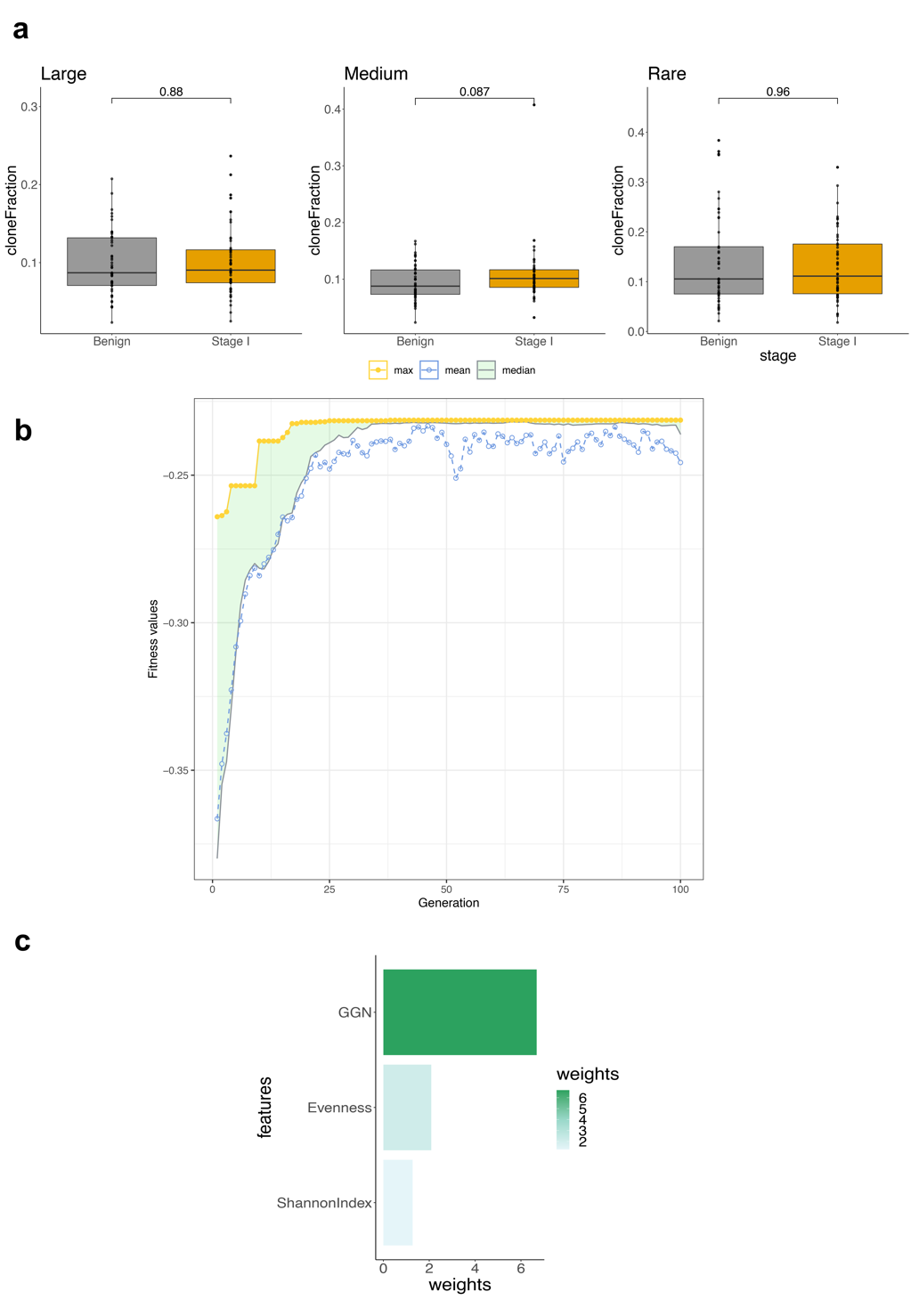


**Figure. S5. The TCR clone type fraction between benign and stage I groups and the construction of the TCRnodseek model**

(a) a comparison of non-significantly different TCR clone types between the different groups in our study. (b) a genetic algorithm with 100 generations is applied to optimize the parameters of the SVM model. (c) The weights of the features in the last TCRnodseek model are shown in a bar plot.


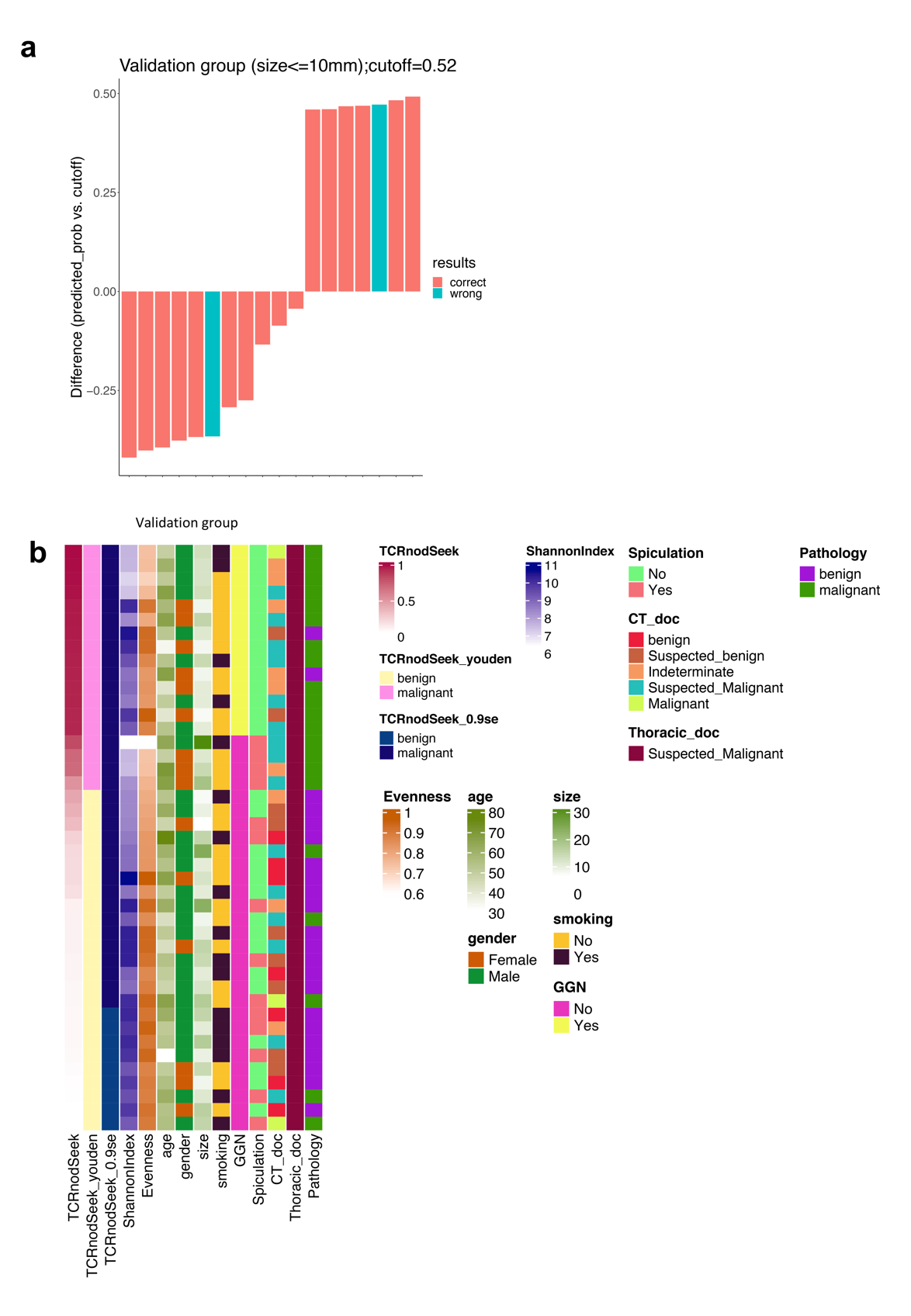


Figure. S6. Correlation between TCRnodseek and clinical characteristics

(a) The waterfall plot illustrates the TCRnodseek function in nodules of size (
<10mm). (b) Heatmaps illustrating the correlation between TCRnodseek and clinical features





Figure. S7. Boxplot showing public data in immunoSEQ Analyzer

(abc) Three datasets demonstrate TCR diversity and different distributions among different conditions. (d) The distribution of TCR diversity in our study. (e) combining the three datasets and other datasets into one plot.


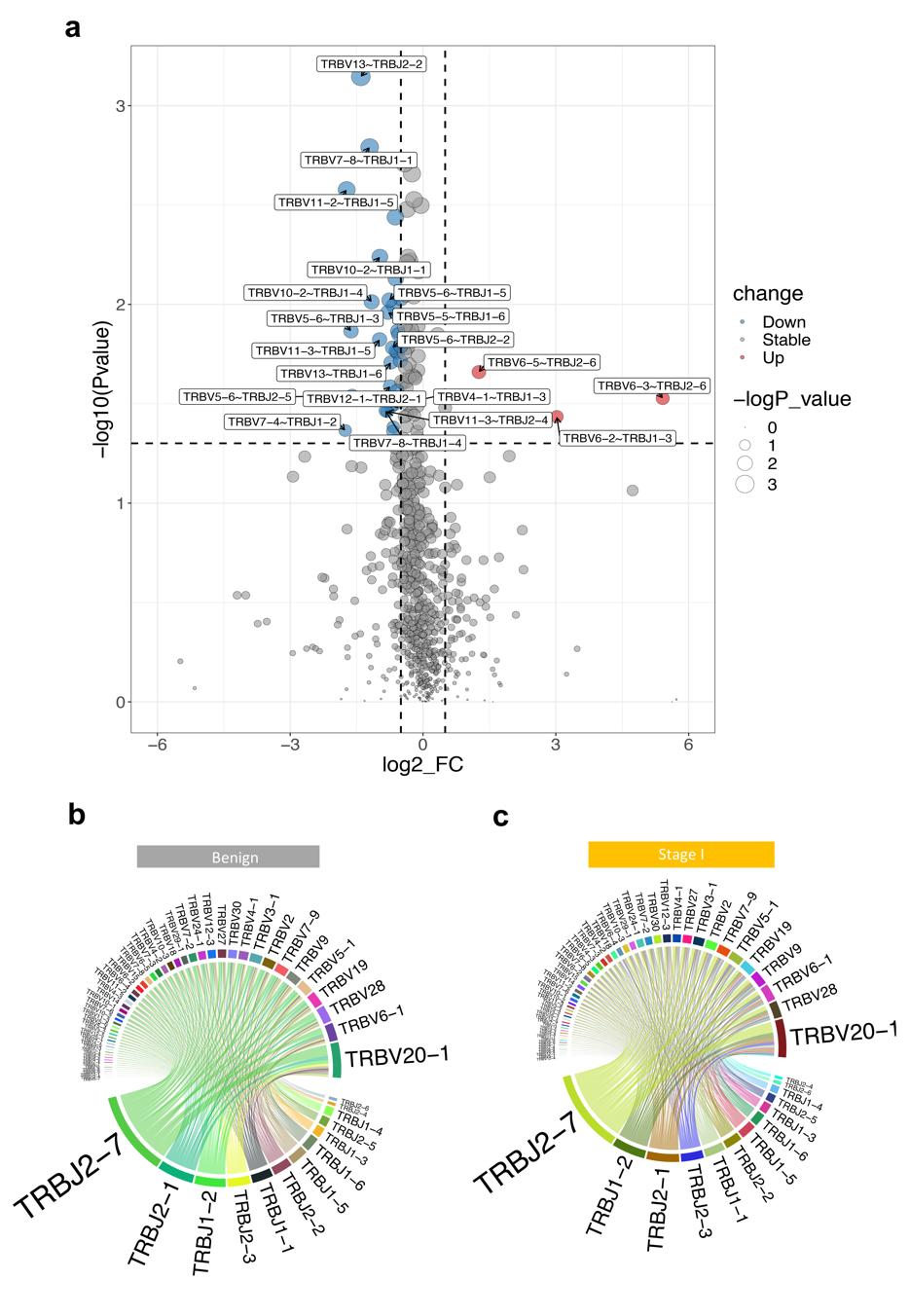


Figure. S8. Usage of TCR Vβ–Jβ gene pairs in enrolled subjects

(a) A volcano plot is used to illustrate the differences between benign and malignant groups in terms of TCR Vβ–Jβ gene pairs usage frequency (cut off of p-value is 0.05; cut off of log2 Fold change is 0.5). TCR-β (TRB) Vβ–Jβ gene pairs in the peripheral blood TCR repertoires of benign and malignant cancer patients. For the benign group (b) and (c) the malignant group, all TCR Vβ–Jβ gene pairs are shown in the chord diagram.

Table S1. Clinical information of main enrolled subjects

| **Features** | |  | **Discover group** | | | | | | | | **Validation group** | | |
| --- | --- | --- | --- | --- | --- | --- | --- | --- | --- | --- | --- | --- | --- |
|  | | **Overall　(N=99)** | **Benign(N=27)** | | | | **Malignant(N=29)** | | **P-value** | | **Benign(N=22)** | **Malignant(N=21)** | **P-value** |
| **Age Mean (SD)** | | 55.5 (9.46) | 57.0 (10.1) | | | | 55.7 (8.80) | | 0.768 | | 54.0 (9.21) | 55.0 (10.1) | 0.679 |
| **Gender** | |  |  | | | |  | |  | |  |  |  |
| **Female** | | 47 (47.5%) | 16 (59.3%) | | | | 16 (55.2%) | | 0.793 | | 7 (31.8%) | 8 (38.1%) | 0.755 |
| **Male** | | 52 (52.5%) | 11 (40.7%) | | | | 13 (44.8%) | |  | | 15 (68.2%) | 13 (61.9%) |  |
| **Stage** | |  |  | | | |  | |  | |  |  |  |
| **Benign** | | 49 (49.5%) | 27 (100%) | | | | 0 (0%) | | - | | 22 (100%) | 0 (0%) | - |
| **IA1** | | 13 (13.1%) | 0 (0%) | | | | 7 (24.1%) | |  | | 0 (0%) | 6 (28.6%) |  |
| **IA2** | | 29 (29.3%) | 0 (0%) | | | | 16 (55.2%) | |  | | 0 (0%) | 13 (61.9%) |  |
| **IA3** | | 7 (7.1%) | 0 (0%) | | | | 6 (20.7%) | |  | | 0 (0%) | 0 (0%) |  |
| **IB** | | 1 (1.0%) | 0 (0%) | | | | 0 (0%) | |  | | 0 (0%) | 1 (4.8%) |  |
| **Family history** | |  |  | | | |  | |  | |  |  |  |
| **No** | | 82 (82.8%) | 22 (81.5%) | | | | 22 (75.9%) | | 0.748 | | 18 (81.8%) | 20 (95.2%) | 0.345 |
| **Yes** | | 17 (17.2%) | 5 (18.5%) | | | | 7 (24.1%) | |  | | 4 (18.2%) | 1 (4.8%) |  |
| **Nodule up** | |  |  | | | |  | |  | |  |  |  |
| **No** | | 29 (29.3%) | 12 (44.4%) | | | | 6 (20.7%) | | 0.086 | | 8 (36.4%) | 3 (14.3%) | 0.162 |
| **Yes** | | 70 (70.7%) | 15 (55.6%) | | | | 23 (79.3%) | |  | | 14 (63.6%) | 18 (85.7%) |  |
| **GGN** | |  |  | | | |  | |  | |  |  |  |
| **No** | | 64 (64.6%) | 23 (85.2%) | | | | 12 (41.4%) | | **<0.001** | | 20 (90.9%) | 9 (42.9%) | **0.001** |
| **Yes** | | 35 (35.4%) | 4 (14.8%) | | | | 17 (58.6%) | |  | | 2 (9.1%) | 12 (57.1%) |  |
| **Spiculation** | |  |  | | | |  | |  | |  |  |  |
| **No** | | 77 (77.8%) | 22 (81.5%) | | | | 26 (89.7%) | | 0.462 | | 15 (68.2%) | 14 (66.7%) | 1 |
| **Yes** | | 22 (22.2%) | 5 (18.5%) | | | | 3 (10.3%) | |  | | 7 (31.8%) | 7 (33.3%) |  |
| **Smoking** | |  |  | | | |  | |  | |  |  |  |
| **No** | | 61 (61.6%) | 18 (66.7%) | | | | 17 (58.6%) | | 0.589 | | 12 (54.5%) | 14 (66.7%) | 0.537 |
| **Yes** | | 38 (38.4%) | 9 (33.3%) | | | | 12 (41.4%) | |  | | 10 (45.5%) | 7 (33.3%) |  |
| **Size Mean (SD)** | | 13.7 (5.27) | 13.7 (4.95) | | | | 15.3 (5.84) | | 0.475 | | 11.9 (3.96) | 13.4 (5.73) | 0.449 |
|  |  | | |  |  |  | |  | |  |  |  |  |
|  |  | | |  |  |  | |  | |  |  |  |  |

Table S2. Sequencing quality control data of main enrolled subjects

| **Sample** | **rawReads** | **cleanReads** | **cleanQ30** | **cleanRatio** | **clonalReads** |
| --- | --- | --- | --- | --- | --- |
| I16 | 9295092 | 9137594 | 0.920249 | 0.98 | 868029 |
| I18 | 9225860 | 9077528 | 0.921437 | 0.98 | 964744 |
| I19 | 6728152 | 6608466 | 0.919462 | 0.98 | 694562 |
| I6 | 8334994 | 8202512 | 0.923164 | 0.98 | 846429 |
| I7 | 6684296 | 6555848 | 0.917653 | 0.98 | 693183 |
| L106 | 6548250 | 6466988 | 0.926493 | 0.99 | 392658 |
| L113 | 6352772 | 6249902 | 0.924869 | 0.98 | 595687 |
| L117 | 8193942 | 8069356 | 0.919424 | 0.98 | 552287 |
| L119 | 6096238 | 5891800 | 0.898021 | 0.97 | 693724 |
| L122 | 6817028 | 6691474 | 0.92079 | 0.98 | 584403 |
| L123 | 7293702 | 7193822 | 0.92612 | 0.99 | 537710 |
| L124 | 9215292 | 9092026 | 0.930479 | 0.99 | 1098800 |
| L125 | 6743172 | 6666344 | 0.92901 | 0.99 | 723518 |
| L126 | 6290306 | 6207828 | 0.926958 | 0.99 | 552398 |
| L127 | 6641974 | 6542820 | 0.921528 | 0.99 | 568016 |
| L131 | 6814756 | 6632302 | 0.909692 | 0.97 | 988374 |
| L145 | 7536822 | 7271854 | 0.897781 | 0.96 | 783778 |
| L28 | 7990310 | 7851258 | 0.9191 | 0.98 | 843108 |
| L38 | 8508766 | 8349428 | 0.917668 | 0.98 | 932689 |
| L39 | 6983136 | 6754004 | 0.901269 | 0.97 | 644937 |
| L40 | 6711632 | 6475442 | 0.896059 | 0.96 | 389760 |
| L45 | 6939788 | 6684688 | 0.893837 | 0.96 | 666975 |
| L52 | 7581814 | 7441806 | 0.918622 | 0.98 | 351668 |
| L55 | 8182048 | 8039340 | 0.919941 | 0.98 | 488554 |
| L57 | 8139828 | 8019292 | 0.923144 | 0.99 | 615317 |
| L62 | 7238194 | 7005814 | 0.900286 | 0.97 | 734571 |
| L64 | 7050734 | 6945524 | 0.923423 | 0.99 | 669788 |
| L73 | 6343758 | 6258148 | 0.926167 | 0.99 | 453449 |
| L74 | 7663228 | 7559416 | 0.927132 | 0.99 | 613679 |
| L83 | 7475584 | 7336378 | 0.920264 | 0.98 | 642243 |
| L84 | 7387818 | 7146184 | 0.899243 | 0.97 | 587698 |
| L97 | 6047418 | 5947686 | 0.924949 | 0.98 | 579167 |
| L98 | 6956656 | 6771178 | 0.907428 | 0.97 | 972234 |
| LM456 | 35246454 | 34998372 | 0.949952 | 0.99 | 2797320 |
| LM461 | 28486382 | 28296368 | 0.953235 | 0.99 | 1984084 |
| LM487 | 36700048 | 36358244 | 0.943423 | 0.99 | 1656230 |
| LM500 | 27503930 | 27353734 | 0.953907 | 0.99 | 2726269 |
| LM502 | 28502016 | 28310248 | 0.950925 | 0.99 | 1981149 |
| LM520 | 42328428 | 42114604 | 0.959924 | 0.99 | 6534048 |
| LM521 | 38793010 | 38523166 | 0.947075 | 0.99 | 3587426 |
| LM534 | 36858648 | 36596144 | 0.947149 | 0.99 | 3774361 |
| LM535 | 29819852 | 29579932 | 0.949294 | 0.99 | 2133064 |
| LM540 | 33352538 | 33154942 | 0.951076 | 0.99 | 1764094 |
| LM550 | 38632250 | 38060788 | 0.937379 | 0.99 | 1767047 |
| LM552 | 36197848 | 35844888 | 0.947094 | 0.99 | 3108493 |
| LM556 | 37535732 | 37332284 | 0.950372 | 0.99 | 2732560 |
| LM558 | 32782894 | 32508480 | 0.943772 | 0.99 | 651093 |
| LM562 | 40340344 | 40052414 | 0.942155 | 0.99 | 4033736 |
| LM563 | 33019616 | 32757884 | 0.946326 | 0.99 | 1433876 |
| LM569 | 33324798 | 33087536 | 0.948672 | 0.99 | 1925937 |
| LM570 | 36008618 | 35797112 | 0.952136 | 0.99 | 2930015 |
| LM578 | 26308626 | 26104702 | 0.950844 | 0.99 | 2054464 |
| LM579 | 33939160 | 33657924 | 0.949731 | 0.99 | 2772806 |
| LM584 | 33268598 | 32990850 | 0.948179 | 0.99 | 1749116 |
| LM596 | 39134986 | 38862330 | 0.947825 | 0.99 | 2349435 |
| LM610 | 37804136 | 37446582 | 0.948885 | 0.99 | 2846081 |
| LM622 | 35880654 | 35595980 | 0.945458 | 0.99 | 1559997 |
| LM627 | 38227560 | 37995028 | 0.949014 | 0.99 | 2238241 |
| LM629 | 38637654 | 38290118 | 0.942508 | 0.99 | 1768913 |
| LM632 | 38608302 | 38358598 | 0.947905 | 0.99 | 2311561 |
| LM633 | 32836734 | 32601068 | 0.949111 | 0.99 | 2825034 |
| LM641 | 38836484 | 38568388 | 0.948222 | 0.99 | 2896592 |
| LM646 | 34455128 | 34161238 | 0.945382 | 0.99 | 1662969 |
| LM656 | 31193718 | 30968266 | 0.951296 | 0.99 | 2452384 |
| LM657 | 40057828 | 39770304 | 0.946031 | 0.99 | 2321662 |
| LM659 | 32640978 | 32435944 | 0.951105 | 0.99 | 2667513 |
| LM675 | 35848752 | 35621566 | 0.950116 | 0.99 | 2621160 |
| LM695 | 26712170 | 26467652 | 0.946957 | 0.99 | 1719495 |
| LM696 | 32629754 | 32372598 | 0.947163 | 0.99 | 2181241 |
| LM705 | 34054074 | 33833788 | 0.95076 | 0.99 | 2967396 |
| LM708 | 40104320 | 39766592 | 0.944299 | 0.99 | 2776277 |
| LM711 | 40063782 | 39704430 | 0.943581 | 0.99 | 1951949 |
| LM734 | 32994004 | 32789094 | 0.950433 | 0.99 | 1415127 |
| LM735 | 35716724 | 35446060 | 0.945519 | 0.99 | 1784021 |
| LM736 | 34091966 | 33837844 | 0.952091 | 0.99 | 4017911 |
| LM737 | 39799408 | 39475618 | 0.943735 | 0.99 | 2127032 |
| LM739 | 32656302 | 32487792 | 0.954463 | 0.99 | 2546967 |
| LM741 | 37834260 | 37593910 | 0.947691 | 0.99 | 2953155 |
| LM745 | 27272566 | 27114646 | 0.952569 | 0.99 | 1518261 |
| LM747 | 29955652 | 29749562 | 0.949622 | 0.99 | 2467778 |
| LM754 | 39394050 | 39096192 | 0.946402 | 0.99 | 2359258 |
| LM758 | 36431844 | 36079070 | 0.942589 | 0.99 | 1785575 |
| LM761 | 35131240 | 34869618 | 0.948898 | 0.99 | 3022926 |
| LM764 | 32799302 | 32605634 | 0.952712 | 0.99 | 3297960 |
| LM771 | 38196356 | 37932366 | 0.947125 | 0.99 | 1917951 |
| LM784 | 43951690 | 43632002 | 0.945225 | 0.99 | 2397807 |
| LM786 | 33268784 | 33029286 | 0.948913 | 0.99 | 2391543 |
| LM789 | 42584378 | 42166946 | 0.938882 | 0.99 | 1734244 |
| LM790 | 38430534 | 38156220 | 0.946958 | 0.99 | 1946899 |
| N100 | 51248708 | 50444662 | 0.931901 | 0.98 | 3889107 |
| N114 | 40051990 | 39511328 | 0.930889 | 0.99 | 2287929 |
| N24 | 49671314 | 48917048 | 0.9329 | 0.98 | 2811288 |
| N33 | 46137372 | 45571072 | 0.9368 | 0.99 | 3827337 |
| N34 | 33301816 | 32616778 | 0.927465 | 0.98 | 1769340 |
| N58 | 47514722 | 46900204 | 0.933699 | 0.99 | 3802216 |
| N59 | 47353940 | 46625222 | 0.93245 | 0.98 | 2809596 |
| N60 | 30863956 | 30171494 | 0.925176 | 0.98 | 1716186 |
| N85 | 49698518 | 48988242 | 0.931667 | 0.99 | 3259882 |
| N95 | 55517646 | 54784342 | 0.944168 | 0.99 | 4342605 |
| N27 | 52583984 | 51877472 | 0.931792 | 0.99 | 2018125 |
| N28 | 45855250 | 45176838 | 0.935282 | 0.99 | 3048438 |
| N32 | 41943728 | 41348814 | 0.933178 | 0.99 | 2761111 |
| N63 | 43644850 | 42951770 | 0.934094 | 0.98 | 2683959 |
| N99 | 42671616 | 42054596 | 0.935645 | 0.99 | 4422975 |
| HL210 | 57881980 | 57291050 | 0.936745 | 0.99 | 4980280 |
| HL214 | 44587354 | 43896618 | 0.93872 | 0.98 | 5501579 |
| HL224 | 46127326 | 45355278 | 0.934988 | 0.98 | 4622983 |
| HL219 | 48091724 | 47270680 | 0.934767 | 0.98 | 5671251 |
| HL190 | 35005576 | 34312176 | 0.926539 | 0.98 | 2182985 |

Data S1. (separate file)

Clone Fraction, annotation of all candidate TCR clones and HLA genotyping data

Data S2. (separate file)

The raw data of common motifs identified by MEME tool

Data S3. (separate file)

TCR diversity information of main enrolled subjects

Data S4. (separate file)

V/J gene segment usage data of main enrolled subjects

**Reference**

1 Liu, Y.-Y. *et al.* Characteristics and prognostic significance of profiling the peripheral blood T-cell receptor repertoire in patients with advanced lung cancer. *Int J Cancer*. **145**, 1423-1431, (2019).

2 Chen, S., Zhou, Y., Chen, Y. & Gu, J. fastp: an ultra-fast all-in-one FASTQ preprocessor. *Bioinformatics*. **34**, i884-i890, (2018).

3 Bolotin, D. A. *et al.* MiXCR: software for comprehensive adaptive immunity profiling. *Nat Methods*. **12**, 380-381, (2015).

4 Shugay, M. *et al.* VDJtools: Unifying Post-analysis of T Cell Receptor Repertoires. *PLoS Comput Biol*. **11**, e1004503, (2015).

5 Chung, K. *et al.* Brock malignancy risk calculator for pulmonary nodules: validation outside a lung cancer screening population. *Thorax*. **73**, 857-863, (2018).

6 Swensen, S. J. *et al.* The probability of malignancy in solitary pulmonary nodules. Application to small radiologically indeterminate nodules. *Arch Intern Med*. **157**, 849-855, (1997).

7 Gould, M. K., Ananth, L. & Barnett, P. G. A clinical model to estimate the pretest probability of lung cancer in patients with solitary pulmonary nodules. *Chest*. **131**, 383-388, (2007).

8 Chen, Y. *et al.* High-throughput T cell receptor sequencing reveals distinct repertoires between tumor and adjacent non-tumor tissues in HBV-associated HCC. *Oncoimmunology*. **5**, e1219010, (2016).

9 Stalika, E. *et al.* Skewing of the T cell receptor gene repertoire and public clonotypes in cytotoxic T cells of patients with chronic idiopathic neutropenia: a role for antigen selection in disease development. *Blood*. **120**, 831, (2012).

10 Tickotsky, N. *et al.* McPAS-TCR: a manually curated catalogue of pathology-associated T cell receptor sequences. *Bioinformatics*. **33**, 2924-2929, (2017).

11 Chronister, W. D. *et al.* TCRMatch: Predicting T-Cell Receptor Specificity Based on Sequence Similarity to Previously Characterized Receptors. *Front Immunol*. **12**, 640725, (2021).

12 Bagaev, D. V. *et al.* VDJdb in 2019: database extension, new analysis infrastructure and a T-cell receptor motif compendium. *Nucleic Acids Res*. **48**, D1057-D1062, (2020).

13 Bailey, T. L. & Elkan, C. Fitting a mixture model by expectation maximization to discover motifs in biopolymers. *Proc Int Conf Intell Syst Mol Biol*. **2**, 28-36, (1994).

14 Harjanto, S., Ng, L. F. P. & Tong, J. C. Clustering HLA class I superfamilies using structural interaction patterns. *PloS One*. **9**, e86655, (2014).

15 Lu, T. *et al.* Deep learning-based prediction of the T cell receptor–antigen binding specificity. *Nat Mach Intell*. **3**, 864-875, (2021).
